# Nonlinear Relationship of Weight‐Adjusted Waist Index With Self‐Reported Trouble Sleeping and the Mediating Role of Depression

**DOI:** 10.1155/jobe/8895676

**Published:** 2026-11-02

**Authors:** Yuhan Song, Zhaoxiang Wang, Xuejing Shao, Shao Zhong

**Affiliations:** ^1^ Department of Endocrinology Affiliated Kunshan Hospital of Jiangsu University Kunshan Jiangsu 215300 China ujs.edu.cn; ^2^ Department of Endocrinology Affiliated Wujin Hospital of Jiangsu University Wujin Clinical College of Xuzhou Medical University Changzhou Jiangsu 213017 China

**Keywords:** depression, nonlinear relationship, obesity paradox, trouble sleeping, weight-adjusted-waist index

## Abstract

**Purpose:**

The weight‐adjusted waist index (WWI) is a novel measure for obesity that appears to outperform body mass index (BMI) and waist circumference (WC) in assessing lean and fat mass. Our aim was to explore the possible link between WWI and trouble sleeping.

**Methods:**

This cross‐sectional study included 30,999 adults who participated in the National Health and Nutrition Examination Survey (NHANES) database. The WWI was calculated as WC (cm) divided by the square root of weight (kg). Trouble sleeping is based on a self‐reported medical history. The relationship between WWI and trouble sleeping was explored through logistic regression, smooth curve fitting, mediation analysis, and receiver operating characteristic (ROC) analysis.

**Results:**

As WWI levels increase, self‐reported trouble sleeping becomes more prevalent (20.96% vs. 25.97% vs. 30.26% vs. 37.15%, *p* < 0.001). Multivariable logistic regression analysis showed that higher WWI was associated with a greater likelihood of self‐reported trouble sleeping (OR [95%CI]: 1.10 [1.03–1.18], *p* = 0.005). ROC analysis showed modest discrimination for WWI, with an area under the curve (AUC) of 0.6041. Smooth curve fitting uncovered a nonlinear relationship, with a breakpoint at 11.04. Additionally, depression partly mediated the WWI‐trouble sleeping connection.

**Conclusions:**

Higher WWI was associated with a higher prevalence of self‐reported trouble sleeping. Although WWI may serve as a practical anthropometric indicator, its modest discriminatory ability and the cross‐sectional study design warrant cautious interpretation.

## 1. Introduction

Sleep health is emerging as a significant public health issue [1]. As societal pace quickens, approximately one‐third of the population reports dissatisfaction with sleep, experiencing difficulties in falling or staying asleep, or waking up prematurely [2, 3]. Trouble sleeping, including obstructive sleep apnea, concerns about sleep quality (such as insufficient sleep, sleep duration, and insomnia), along with a combination of other sleep issues, is progressively affecting US adults [4, 5]. Trouble sleeping often coexists with other physical or mental health conditions, such as obesity, depression, and cardiovascular diseases [6–8].

Body mass index (BMI, kg/m^2^) is a commonly used tool for assessing obesity, but it has increasingly come under scrutiny in recent years for its inability to effectively differentiate between types of fat or distribution, as well as muscle mass [9]. On the other hand, the clinical use of advanced technologies such as computed tomography (CT) and magnetic resonance imaging (MRI) is limited by high costs, restricted accessibility, long operation times, and radiation exposure. The “obesity paradox” has been observed to various extents [9]. Visceral fat more closely correlates with poor metabolic profiles, often tied to abdominal obesity [10]. Waist circumference (WC, cm) is currently recognized as a better obesity‐related indicator because of its strong correlation with abdominal fat [11]. Consequently, Park et al. proposed the weight‐adjusted‐waist index (WWI) in 2018, defined as WC divided by the square root of body weight, as a novel and convenient obesity indicator [12]. It capitalizes on the advantages of WC while reducing its correlation with BMI [12]. Studies have shown that WWI offers a more detailed differentiation between fat and muscle mass, focusing primarily on abdominal obesity independent of body weight [13–15].

Obesity is closely associated with sleep disorders through multiple pathways involving respiration, inflammation, metabolism, and psychological factors. Conversely, poor sleep quality may promote weight gain and central obesity by disrupting appetite regulation, energy metabolism, and related health behaviors [8, 16–19]. However, evidence remains limited regarding the association between the WWI and trouble sleeping. To fill this gap, we carried out a cross‐sectional analysis utilizing data from the National Health and Nutrition Examination Survey (NHANES) spanning 2007–2018 to explore the role of WWI in self‐reported trouble sleeping and to uncover possible mechanisms of association.

## 2. Materials and Methods

### 2.1. Study Population

The NHANES, conducted by the National Center for Health Statistics (NCHS), is an ongoing national survey designed to assess the health and nutritional status of the US population (https://wwwn.cdc.gov/nchs/nhanes) [20]. It employs a complex multistage stratified sampling method. We incorporated data from six cycles of NHANES (2007–2008, 2009–2010, 2011–2012, 2013–2014, 2015–2016, 2017–2018), excluding individuals under 20 years old, those who were pregnant, and those lacking WWI and sleep questionnaire data. Ultimately, our study population consisted of 30, 999 participants. All participants provided written informed consent, and the NHANES protocol was approved by the NCHS Research Ethics Review Board.

### 2.2. Measures of Exposure and Outcome

WWI was calculated as WC (cm) divided by the square root of body weight (kg). On the other hand, self‐reported trouble sleeping was assessed by a healthcare professional using the NHANES question: “Have you ever told a doctor or other health professional that you have trouble sleeping?” with responses “Yes,” “No,” “Refused,” and “Don’t know.” “Refused” and “Don’t know” were treated as missing values [8, 21–23]. This outcome variable reflects participants’ self‐reported history of reporting trouble sleeping to a healthcare professional rather than a clinically confirmed diagnosis of a sleep disorder.

### 2.3. Covariates

Covariates were selected a priori based on established or biologically plausible associations with adiposity, sleep problems, and cardiometabolic health, as well as their availability and standardized assessment in NHANES. The selected variables included age, gender, and race, marital status, household income, education level, smoking status, presence of diabetes, hypertension, cardiovascular diseases, and depression. Among other factors, BMI, glycated hemoglobin (HbA1c, %), alanine transaminase (ALT, U/L), aspartate transaminase (AST, U/L), gamma‐glutamyl transferase (GGT, U/L), triglycerides (TG, mmol/L), total cholesterol (TC, mmol/L), high‐density lipoprotein cholesterol (HDL‐c, mmol/L), low‐density lipoprotein cholesterol (LDL‐c, mmol/L), serum creatinine (Scr, μmol/L), and the estimated glomerular filtration rate (eGFR, mL/min/1.73 m^2^) were also considered. Based on the age, gender, race, and Scr levels, the eGFR was estimated using the CKD‐EPI formula [24]. The diagnosis of diabetes and hypertension is based on self‐reported medical history, whereas cardiovascular diseases require participants to report previous heart attacks, strokes, heart failure, coronary artery disease, or angina. Depression was evaluated based on the Patient Health Questionnaire (PHQ‐9), a nine‐question tool assessing depressive symptom frequency over the past 2 weeks [25]. Responses were rated from 0 to 3, corresponding to “not at all,” “several days,” “more than half the days,” and “nearly every day.” This tool aligns with DSM‐IV criteria for depression, with scores ranging from 0 to 27. Participants with a score of 10 or higher were identified as having major depression [25].

### 2.4. Statistical Analysis

Population‐weighted statistical descriptions were employed. Continuous variables were presented as means with standard errors and categorical measures as percentages. Differences among groups were assessed using weighted *t*‐tests (continuous variables) and weighted chi‐square tests (categorical variables). Missing data are summarized in Supporting Information: Table S1, including the number and percentage of missing observations for each variable. We assumed that the data were missing at random (MAR). Missing values were imputed using the random forest‐based missForest algorithm implemented in the R package missForest. This nonparametric approach iteratively predicts missing values using random forest models based on the available information for the remaining variables. Each random forest comprised 100 trees, and the imputation procedure was repeated for 10 iterations. We constructed three separate logistic regression models to examine the independent relationship between WWI and trouble sleeping. Model 1 did not adjust for any covariates; Model 2 adjusted for age, gender, and race; and Model 3 included adjustments for age, gender, and race, marital status, household income, education level, smoking status, diabetes, hypertension, cardiovascular diseases, depression, HbA1c, ALT, AST, GGT, TG, TC, LDL‐c, HDL‐c, Scr, and eGFR. Smooth curve fitting analysis was conducted to investigate potential nonlinear associations. When nonlinear relationships were detected, a two‐piecewise linear regression model was used to establish intervals and detect threshold effects. Receiver operating characteristic (ROC) analysis and decision curve analysis (DCA) were used to compare the performance of WWI with other obesity indicators in relation to trouble sleeping. Subgroup analyses were also conducted based on age, gender, BMI, race, diabetes, hypertension, and cardiovascular diseases to examine the impact of WWI on trouble sleeping. Finally, considering depression as a potential pathway linking WWI to trouble sleeping, we conducted a mediation analysis [26, 27]. WWI was specified as the exposure, depression as the mediator, and trouble sleeping as the outcome. The total effect was estimated without adjustment for depression, whereas the direct and indirect effects were estimated after its inclusion. All models were adjusted for prespecified covariates and sampling weights. The indirect effect was estimated using nonparametric bootstrap resampling with 1000 replications, and bias‐corrected 95% confidence intervals were calculated. All statistical analyses were conducted using Stata 18.0, R software, and EmpowerStats (https://www.empowerstats.com, X&Y Solutions, Inc., Boston, MA, USA). A multistage survey design incorporating sample weights from six cycles was employed in the statistical analysis, in accordance with the Centers for Disease Control and Prevention (CDC) guidelines. A *p* value < 0.05 (two‐tailed) was deemed statistically significant.

## 3. Results

### 3.1. Baseline Characteristics

There were 30,999 individuals analyzed in total, with an average age of 47.40 years (Table 1). In terms of gender, 48.98% of the participants were males and 51.02% were females. Racial identifications included 66.29% non‐Hispanic whites, 11.14% non‐Hispanic blacks, 8.55% Mexican Americans, 5.93% Other Hispanics, and 8.09% other races. In comparison to the non‐trouble sleeping group, those with trouble sleeping are older, have a higher proportion of females, have lower incomes, and are more likely to smoke, suffer from hypertension, diabetes, cardiovascular diseases, and depression (*p* < 0.001). Moreover, trouble sleeping group has elevated levels of BMI, WC, HbA1c, ALT, AST, GGT, TG, and TC (*p* < 0.05). On the other hand, the proportion of married individuals and eGFR levels were decreased among those with trouble sleeping (*p* < 0.001). Note that WWI levels were higher in trouble sleeping group than in the non‐trouble sleeping group (11.16 ± 0.02 vs. 10.90 ± 0.01, *p* < 0.001).

**TABLE 1:** Baseline characteristics of participants according to sleep status, weighted.

	Overall (*N* = 30,999)	Non‐trouble sleeping (*N* = 23,012)	Trouble sleeping (*N* = 7987)	*p* value
Age (years)	47.40 ± 0.23	46.06 ± 0.25	50.85 ± 0.28	< 0.001
Male gender (%)	48.98	51.75	41.81	< 0.001
Race (%)				< 0.001
Mexican American	8.55	9.88	5.12	
Non‐Hispanic Black	11.14	11.60	9.94	
Non‐Hispanic White	66.29	63.21	74.28	
Other Hispanic	5.93	6.53	4.37	
Other races	8.09	8.78	6.30	
Married (%)	55.09	56.12	52.42	< 0.001
Annual household income (under $20,000) (%)	14.04	13.18	16.26	< 0.001
Education level (above high school) (%)	61.22	60.86	62.14	0.162
Smokers (%)	44.44	40.89	53.64	< 0.001
Diabetes (%)	9.60	7.94	13.90	< 0.001
Hypertension (%)	31.76	26.83	44.52	< 0.001
Cardiovascular diseases (%)	8.22	6.39	12.95	< 0.001
Depression (%)	7.96	4.20	17.50	< 0.001
BMI (kg/m^2^)	28.99 ± 0.09	28.56 ± 0.09	30.12 ± 0.14	< 0.001
WC (cm)	99.25 ± 0.23	98.02 ± 0.23	102.46 ± 0.35	< 0.001
HbA1c (%)	5.64 ± 0.01	5.60 ± 0.01	5.74 ± 0.01	< 0.001
ALT (U/L)	25.27 ± 0.15	25.03 ± 0.16	25.89 ± 0.30	0.010
AST (U/L)	25.23 ± 0.12	24.96 ± 0.12	25.92 ± 0.26	0.001
GGT (U/L)	27.96 ± 0.28	26.60 ± 0.31	31.45 ± 0.72	< 0.001
TG (mmol/L)	1.40 ± 0.02	1.35 ± 0.01	1.50 ± 0.03	< 0.001
TC (mmol/L)	5.00 ± 0.01	4.98 ± 0.01	5.04 ± 0.02	0.002
LDL‐c (mmol/L)	2.94 ± 0.01	2.95 ± 0.01	2.94 ± 0.02	0.791
HDL‐c (mmol/L)	1.38 ± 0.01	1.38 ± 0.01	1.39 ± 0.01	0.110
Scr (μmol/L)	77.98 ± 0.24	77.78 ± 0.25	78.49 ± 0.46	0.144
eGFR (ml/min/1.73 m^2^)	94.78 ± 0.31	96.22 ± 0.34	91.05 ± 0.38	< 0.001
WWI	10.97 ± 0.01	10.90 ± 0.01	11.16 ± 0.02	< 0.001

*Note:* ALT, alanine transaminase; AST, aspartate transaminase; HbA1c, glycated hemoglobin; Scr, serum creatinine; TG, triglyceride.

Abbreviations: BMI, body mass index; eGFR, estimated glomerular filtration rate; GGT, gamma‐glutamyl transferase; HDL‐c, high‐density lipoprotein cholesterol; LDL‐c, low‐density lipoprotein cholesterol; TC, total cholesterol; WC, waist circumference; WWI, weight‐adjusted‐waist index.

### 3.2. Clinical Features of the Participants According to WWI Quartiles

A quartile‐based distribution of WWI levels was used to divide participants into four groups (Table 2). High WWI quartile participants were older, more often female, married, had annual household incomes under $20,000, and were more likely to smoke and have diabetes, hypertension, cardiovascular diseases, and depression (*p* < 0.001). BMI, WC, HbA1c, ALT, AST, GGT, TG, TC, LDL‐c, and Scr levels were also significantly elevated (*p* < 0.05). The proportion of those with education above high school as well as levels of eGFR and HDL‐c was reduced (*p* < 0.001). The most important, as WWI levels increased progressively, prevalence of trouble sleeping also showed a gradual increase (20.96% vs. 25.97% vs. 30.26% vs. 37.15%, *p* < 0.001).

**TABLE 2:** Baseline characteristics of participants according to WWI quartiles, weighted.

	Quartile 1	Quartile 2	Quartile 3	Quartile 4	*p* value
Age (years)	37.28 ± 0.31	46.08 ± 0.25	51.92 ± 0.30	57.94 ± 0.30	< 0.001
Male gender (%)	58.44	52.70	47.29	33.10	< 0.001
Race (%)					< 0.001
Mexican American	5.24	8.80	10.80	10.30	
Non‐Hispanic Black	14.52	10.07	9.86	9.24	
Non‐Hispanic White	66.52	65.82	64.74	68.32	
Other Hispanic	5.13	6.05	6.79	5.91	
Other races	8.60	9.26	7.81	6.23	
Married (%)	47.79	60.02	60.61	52.72	< 0.001
Annual household income (under $20,000) (%)	11.34	11.08	14.56	21.00	< 0.001
Education level (above high school) (%)	70.15	63.92	56.57	50.70	< 0.001
Smokers (%)	38.72	44.15	48.14	48.57	< 0.001
Diabetes (%)	1.91	5.91	11.36	22.92	< 0.001
Hypertension (%)	13.31	28.37	39.02	53.40	< 0.001
Cardiovascular diseases (%)	2.08	5.65	10.64	17.23	< 0.001
Depression (%)	5.90	6.81	8.26	11.91	< 0.001
BMI (kg/m^2^)	24.84 ± 0.08	28.20 ± 0.08	30.50 ± 0.11	34.04 ± 0.15	< 0.001
WC (cm)	85.79 ± 0.20	96.88 ± 0.18	104.47 ± 0.22	114.98 ± 0.32	< 0.001
HbA1c (%)	5.31 ± 0.01	5.54 ± 0.01	5.76 ± 0.02	6.07 ± 0.02	< 0.001
ALT (U/L)	23.11 ± 0.24	26.07 ± 0.32	26.87 ± 0.28	25.43 ± 0.34	< 0.001
AST (U/L)	24.77 ± 0.17	25.07 ± 0.23	25.80 ± 0.27	25.41 ± 0.29	0.019
GGT (U/L)	22.44 ± 0.40	27.83 ± 0.47	31.11 ± 0.78	32.16 ± 0.58	< 0.001
TG (mmol/L)	1.12 ± 0.03	1.39 ± 0.02	1.54 ± 0.03	1.62 ± 0.03	< 0.001
TC (mmol/L)	4.80 ± 0.02	5.08 ± 0.02	5.12 ± 0.02	5.02 ± 0.02	< 0.001
LDL‐c (mmol/L)	2.80 ± 0.02	3.03 ± 0.02	3.04 ± 0.02	2.93 ± 0.03	< 0.001
HDL‐c (mmol/L)	1.48 ± 0.01	1.38 ± 0.01	1.32 ± 0.01	1.31 ± 0.01	< 0.001
Scr (μmol/L)	79.20 ± 0.35	77.26 ± 0.39	77.64 ± 0.40	77.61 ± 0.46	< 0.001
eGFR (ml/min/1.73 m^2^)	101.42 ± 0.41	96.55 ± 0.41	92.02 ± 0.38	86.48 ± 0.43	< 0.001
Trouble sleeping (%)	20.96	25.97	30.26	37.15	< 0.001

*Note:* ALT, alanine transaminase; AST, aspartate transaminase; HbA1c, glycated hemoglobin; Scr, serum creatinine; TG, triglyceride.

Abbreviations: BMI, body mass index; eGFR, estimated glomerular filtration rate; GGT, gamma‐glutamyl transferase; HDL‐c, high‐density lipoprotein cholesterol; LDL‐c, low‐density lipoprotein cholesterol; TC, total cholesterol; WC, waist circumference.

### 3.3. The Association Between WWI and Trouble Sleeping

Table 3 illustrates a positive association between WWI and the prevalence of trouble sleeping across models 1, 2, and 3. Participants with higher WWI had greater odds of self‐reported trouble sleeping after full adjustment (OR [95%CI]: 1.10 [1.03–1.18], *p* = 0.005). When categorizing WWI into quartiles, this association retained its statistical significance, with those in the highest WWI quartile had 28% higher odds of reporting trouble sleeping than those in the lowest quartile (OR [95%CI]: 1.28 [1.09–1.49], *p* = 0.002). Smooth curve fitting indicates a nonlinear relationship between WWI levels and trouble sleeping (Figure 1). Employing a segmented linear regression model, a breakpoint (K) at 11.04 was determined for WWI levels within the population (Table 4). To the left of the threshold, as WWI levels increase, the prevalence of trouble sleeping rises, with an OR of 1.23 and a 95%CI of 1.09–1.40. Beyond the breakpoint, to the right, the relationship between WWI levels and trouble sleeping tends toward saturation. Finally, results from DCA and ROC assessments reveal that WWI had a numerically higher area under the curve (AUC) than BMI and WC. In ROC analysis, the AUC for WWI is 0.6041, compared to 0.5593 for BMI, and 0.5890 for WC (Figure 2a,b).

**TABLE 3:** Logistic regression analysis results of WWI and trouble sleeping.

	Model 1 OR (95%CI) *p* value	Model 2 OR (95%CI) *p* value	Model 3 OR (95%CI) *p* value
*Continuous*			
WWI	1.42 (1.37, 1.46) < 0.001	1.29 (1.24, 1.34) < 0.001	1.10 (1.03, 1.18) 0.005

*Categories*			
Quantile 1	reference	reference	reference
Quantile 2	1.32 (1.22, 1.43) < 0.001	1.26 (1.16, 1.36) < 0.001	1.18 (1.04, 1.35) 0.013
Quantile 3	1.60 (1.48, 1.72) < 0.001	1.44 (1.33, 1.56) < 0.001	1.20 (1.04, 1.39) 0.012
Quantile 4	2.18 (2.02, 2.35) < 0.001	1.77 (1.62, 1.93) < 0.001	1.28 (1.09, 1.49) 0.002
*p* for trend	< 0.001	< 0.001	0.005

*Note:* Model 1: no covariates adjusted. Model 2: adjusted for age, gender, and race. Model 3: adjusted for age, gender, and race, marital status, household income, education level, smoking status, diabetes, hypertension, cardiovascular diseases, depression, HbA1c, ALT, AST, GGT, TG, TC, LDL‐c, HDL‐c, Scr, and eGFR.

Abbreviations: 95% CI, 95% confidence interval; OR, odds ratio.

**FIGURE 1:**
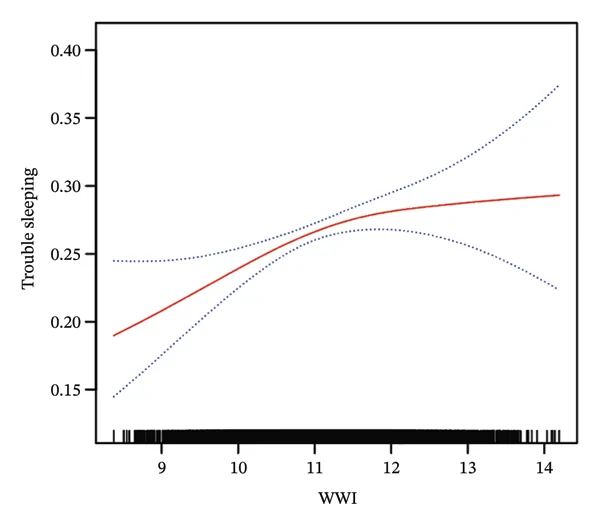
The results of smooth curve fitting analysis.

**TABLE 4:** Threshold effect analysis of WWI on trouble sleeping.

Model	OR (95% CI) *p* value
*Total*	
Breakpoint (K)	11.04
OR1 (< 11.04)	1.23 (1.09, 1.40) 0.001
OR2 (> 11.04)	1.02 (0.92, 1.13) 0.732
OR2/OR1	0.83 (0.69, 0.99) 0.038
*p* for logarithmic likelihood ratio	0.038

*Note:* Adjusted for age, gender, and race, marital status, household income, education level, smoking status, diabetes, hypertension, cardiovascular diseases, depression, HbA1c, ALT, AST, GGT, TG, TC, LDL‐c, HDL‐c, Scr, and eGFR.

**FIGURE 2:**
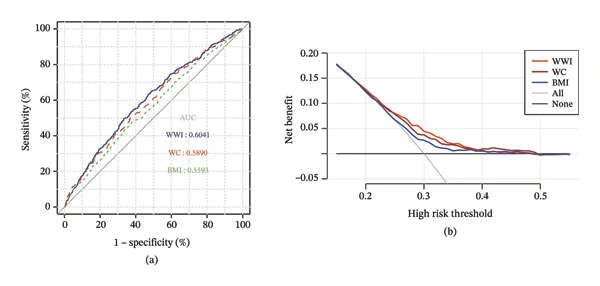
ROC and DCA results.

### 3.4. Subgroup and Mediation Analysis

The cross‐sectional association between WWI and trouble sleeping was stable across various subgroups, showing no significant interaction (Figure 3) (*p* for interaction > 0.05). We also assessed the potential mediation effects in the correlation between WWI and trouble sleeping (Table 5). The exploratory mediation analysis revealed a notable mediating role of depression in the association between WWI and trouble sleeping after adjusting for covariates. The total effect of WWI on trouble sleeping was significant (*β* = 0.021, 95%CI: 0.010–0.038, *p* < 0.001). After accounting for depression, the direct effect of WWI on trouble sleeping remained significant (*β* = 0.019, 95%CI: 0.006–0.033, *p* = 0.008). In addition, the indirect effect through depression was also significant (*β* = 0.002, 95%CI: 0.001–0.008, *p* = 0.010), suggesting that depression mediated this association and accounted for approximately 9.52% (0.002/0.021) of the total effect.

**FIGURE 3:**
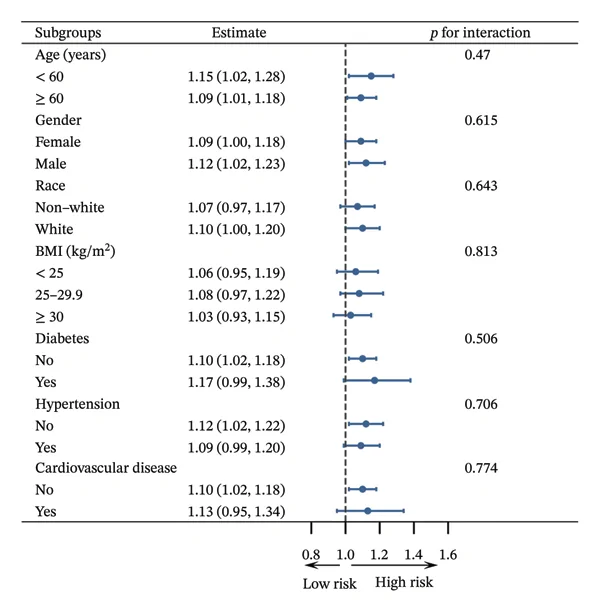
Subgroup analyses.

**TABLE 5:** The mediating effect of depression on the relationship between WWI and trouble sleeping.

Effect types	*β*	95%CI	*p* value
Total effect	0.021	0.010–0.038	< 0.001
Direct effect	0.019	0.006–0.033	0.008
Indirect effect	0.002	0.001–0.008	0.010

*Note:* Adjusted for age, gender, and race, marital status, household income, education level, smoking status, diabetes, hypertension, cardiovascular diseases, HbA1c, ALT, AST, GGT, TG, TC, LDL‐c, HDL‐c, Scr, and eGFR.

## 4. Discussion

Our findings indicate a nonlinear cross‐sectional association between WWI and self‐reported trouble sleeping. Depression may partially mediate this association, suggesting a potential pathway linking higher WWI to greater odds of reporting trouble sleeping.

In epidemiological research, the “obesity paradox” refers to the observation that individuals classified as overweight or obese based on BMI may, in certain populations or disease states, experience health outcomes that are comparable to or even better than those of individuals with a normal BMI. This phenomenon may partly result from the challenge in identifying biologically driven disease risks amid the complex interactions among various anthropometric indicators [9, 28]. Therefore, it is essential to distinguish between lean body mass and fat mass accurately. Prior research also showed that the WWI index effectively distinguishes between fat and muscle mass, with a positive correlation to fat mass and a negative correlation to muscle mass [13–15]. Additionally, compared to emerging obesity indices that rely on relatively complex empirical mathematical models, WWI offers the advantage of being very easy to calculate, facilitating routine screenings in the general population. In a cohort study, WWI outperformed BMI, WC, waist‐to‐height ratio (WHtR), and a body shape index (ABSI) in predicting cardiometabolic morbidity and mortality risk [12]. In our study, ROC and DCA analyses suggested that WWI may provide complementary information for identifying individuals with self‐reported trouble sleeping. Although WWI showed a numerically higher AUC than BMI and WC, all three indices demonstrated modest and comparable discrimination, precluding claims of clear superiority. The incremental net benefit should also be interpreted cautiously, and further longitudinal studies are needed to establish the clinical value of WWI beyond conventional anthropometric measures. Additionally, our study also identified a nonlinear association between WWI and trouble sleeping. The odds of reporting trouble sleeping increased with rising WWI, but this association appeared to plateau at 11.04, suggesting that the impact of WWI on sleep may be most pronounced at low to moderate levels. At higher WWI levels, coexisting metabolic, respiratory, and psychological burdens may saturate the additional risk. This saturation pattern may help refine risk stratification and inform targeted sleep assessment. Epidemiological research cannot confirm whether sleep problems lead to obesity, or obesity causes sleep issues, or if there is a bidirectional relationship [29, 30]. However, the distribution of adipose tissue is associated with the risk of occurrence for certain sleep disorders. Compared to general obesity, abdominal obesity has a stronger correlation with obstructive sleep apnea [31]. Longitudinal studies have indicated that abdominal obesity is an independent risk factor for obstructive sleep apnea [31, 32]. Furthermore, trouble sleeping is associated with mental health issues, such as depression, which are also more prevalent in the population with abdominal obesity [33–36]. In our study, we observed that elevated levels of WWI are associated with a higher prevalence of depression. Previous research also indicates that WWI could serve as a simple anthropometric index for predicting depression [37]. The exploratory mediation analysis identified a statistically significant indirect effect. However, the possibility of shared confounding, reverse causation, and measurement error cannot be excluded, particularly given the cross‐sectional assessment of both variables. Thus, this finding should be interpreted as exploratory rather than as evidence of a causal pathway. The pathophysiological interconnection between abdominal obesity and trouble sleeping encompasses numerous sophisticated mechanisms requiring detailed exploration. Anatomical and respiratory alterations provide the most direct mechanistic pathway linking abdominal obesity to compromised sleep quality [38]. Visceral adiposity‐induced upper airway structural modifications form the pathophysiological foundation of obstructive sleep apnea syndrome [39, 40]. Pharyngeal soft tissue hypertrophy, macroglossia, and pharyngeal luminal reduction collectively impair airway patency throughout sleep periods [39, 40]. Inflammatory processes constitute another fundamental mechanism establishing the link between abdominal obesity and sleep disruption [41]. Visceral adipose tissue demonstrates endocrine activity through the secretion of pro‐inflammatory mediators [42, 43]. These cytokines disrupt physiological sleep–wake regulation through hypothalamic sleep center interference and circadian gene expression modification [44]. Elevated TNF‐α levels characteristic of abdominal obesity demonstrate specific associations with enhanced sleep fragmentation and reduced sleep efficiency [41, 45]. Endocrine and metabolic dysregulation associated with abdominal obesity plays essential roles in sleep pathology. Insulin resistance and glucose metabolic abnormalities influence neurotransmitter biosynthesis and release, particularly affecting sleep‐regulatory serotonin and melatonin systems [46, 47].

We acknowledge several limitations of our study. First, WWI was measured at a single NHANES examination, whereas self‐reported trouble sleeping was assessed retrospectively as a lifetime experience. Thus, sleep problems may have preceded, coincided with, or followed the WWI measurement, and the temporal sequence between exposure and outcome cannot be established. Accordingly, the observed association should be interpreted as cross‐sectional rather than causal. Second, self‐reported histories of trouble sleeping should not be interpreted as validated clinical measures of sleep disturbance, insomnia, or sleep disorder diagnoses. Rather, they reflect participants’ reports of having discussed sleep difficulties with a healthcare professional and may be influenced by healthcare‐seeking behavior, recall bias, and reporting tendencies. Third, physical activity was not adjusted for as a covariate, despite its potential to confound the associations among sleep patterns, obesity, and depression. Future studies with standardized physical activity measures are needed to clarify its confounding or modifying role. Finally, although quartile‐based WWI categorization is commonly used to assess dose–response patterns in epidemiological studies, WWI quartiles do not represent clinically validated thresholds. Unlike WC, which has established standardized cutoffs for abdominal obesity, WWI currently lacks accepted clinical thresholds. Thus, WWI categories should be interpreted as relative, population‐specific risk strata rather than clinically defined groups.

## 5. Conclusion

Our findings indicated WWI may serve as a simple and accessible anthropometric indicator associated with self‐reported trouble sleeping. Nevertheless, its modest and comparable discriminatory performance relative to BMI and WC means that WWI should not be considered a clinically superior or validated predictive tool based on these cross‐sectional findings.

## Funding

This study was supported by the Suzhou Major Diseases Multicenter Clinical Research Program (DZXYJ202419) and the Kunshan High‐Level Health Talent Program Research Project (X25‐016‐101533).

## Ethics Statement

The NHANES protocol was reviewed and approved by the NCHS Research Ethics Review Board (https://www.cdc.gov/nchs/nhanes/about/erb.html). The study was conducted in accordance with the Declaration of Helsinki.

## Consent

The patients/participants provided their written informed consent to participate in this study.

## Conflicts of Interest

The authors declare no conflicts of interest.

## Supporting Information

Additional supporting information can be found online in the Supporting Information section.

## Supporting information


**Supporting Information** Table S1 Missingness of study variables.

## Data Availability

The data that support the findings of this study are openly available in the National Health and Nutrition Examination Survey at https://wwwn.cdc.gov/nchs/nhanes.
