# Supplementary material for: Nonlinear Relationship of Weight‐Adjusted Waist Index With Self‐Reported Trouble Sleeping and the Mediating Role of Depression
Source: J Obes. 2026 Nov 2;2026:8895676. doi: 10.1155/jobe/8895676 (PMC13728453; doi:10.1155/jobe/8895676)
Supplement: Supplementary file 1 [file JOBE-2026-8895676-s001.docx]

| **Supplementary Material Table S1 Missingness of study variable.** | | |
| --- | --- | --- |
| Missing Covariates | Missing, n | Missing, % |
| Marital status | 15 | <0.1% |
| Annual household income | 1669 | 5.3% |
| Education level | 29 | <0.1% |
| Smokers | 19 | <0.1% |
| Diabetes | 21 | <0.1% |
| Hypertension | 37 | 0.1% |
| Cardiovascular diseases | 48 | 1.5% |
| Depression | 2502 | 8.0% |
| BMI | 32 | 0.1% |
| HbA1c | 1213 | 3.9% |
| ALT | 1635 | 5.2% |
| AST | 1651 | 5.3% |
| GGT | 1628 | 5.2% |
| TG | 10980 | 35.4% |
| TC | 1529 | 4.9% |
| LDL-c | 12032 | 38.8% |
| HDL-c | 1529 | 4.9% |
| Scr | 1622 | 5.2% |
| eGFR | 1622 | 5.2% |
